# Innovative Strategies in Tendon Tissue Engineering

**DOI:** 10.3390/pharmaceutics13010089

**Published:** 2021-01-11

**Authors:** Eleonora Bianchi, Marco Ruggeri, Silvia Rossi, Barbara Vigani, Dalila Miele, Maria Cristina Bonferoni, Giuseppina Sandri, Franca Ferrari

**Affiliations:** Department of Drug Sciences, University of Pavia, Viale Taramelli 12, 27100 Pavia, Italy; eleonora.bianchi04@universitadipavia.it (E.B.); marco.ruggeri02@universitadipavia.it (M.R.); silvia.rossi@unipv.it (S.R.); barbara.vigani@unipv.it (B.V.); dalila.miele@unipv.it (D.M.); cbonferoni@unipv.it (M.C.B.); franca.ferrari@unipv.it (F.F.)

**Keywords:** tendon, scaffolds, electrospinning, soft lithography, 3D printing, biological augmentation

## Abstract

The tendon is a highly aligned connective tissue that transmits force from muscle to bone. Each year, more than 32 million tendon injuries have been reported, in fact, tendinopathies represent at least 50% of all sports injuries, and their incidence rates have increased in recent decades due to the aging population. Current clinical grafts used in tendon treatment are subject to several restrictions and there is a significant demand for alternative engineered tissue. For this reason, innovative strategies need to be explored. Tendon replacement and regeneration are complex since scaffolds need to guarantee an adequate hierarchical structured morphology and mechanical properties to stand the load. Moreover, to guide cell proliferation and growth, scaffolds should provide a fibrous network that mimics the collagen arrangement of the extracellular matrix in the tendons. This review focuses on tendon repair and regeneration. Particular attention has been devoted to the innovative approaches in tissue engineering. Advanced manufacturing techniques, such as electrospinning, soft lithography, and three-dimensional (3D) printing, have been described. Furthermore, biological augmentation has been considered, as an emerging strategy with great therapeutic potential.

## 1. Introduction

Tendon disorders are medical conditions, such as ruptures and overuse injuries, and inflammatory and degenerative disorders, such as tendinopathies. In the United States, 33 million musculoskeletal injuries have been reported per year, 50% involving tendon and ligament injuries. Tendon injuries, however, do not only occur in physically active adults and adolescents (especially males), but they also appear among the sedentary population (who conduct moderate physical activity). Next to sports, several intrinsic factors, including body weight, nutrition, and age, may be responsible for tendon injuries. Moreover, genetic diseases that affect connective tissue could have an impact on tendon/ligament. Tendinopathies could be accompanied by inflammation and pain (as tendonitis, peri-tendonitis, and retrocalcaneobursitis), whereas tendinosis and ruptures are caused by intertendinous degeneration without the evidence of inflammatory processes [1]. The incidence of Achilles tendon ruptures (one of the most frequent tendons injured), is up to 1%, typically in 30- to 50-year-old men [2].

Chronic, non-healing tendon injuries frequently require surgical treatment, and despite recent advancements in orthopedic surgery, many common tendon repair techniques yield less than optimal results [3,4,5]. Moreover, healed tendons tend to form scar tissue with different mechanical properties than healthy tendons and are prone to reinjury. Furthermore, at the tendon–bone interface, many common tools used in surgery, such as suture anchors, cannot regenerate the enthesitis, resulting in a high incidence of re-rupture [6,7]. This is due to the peculiarity of the tendon-to-bone interface (TBI), a hybrid complex tissue that serves as shock-absorber of transmission forces between the tendon and the bone. Therefore, alternative surgical procedures should enhance proper tendon healing. This review will focus on tendon structures and their junctions to bones or muscles, the injuries, common treatments to restore limb functions, and the recent strategies of tissue engineering based on three-dimensional (3D) scaffolds and biological augmentation.

## 2. Tendon Structure and Metabolism

Tendons are anatomic structures interposed between muscles and bones. Their function is to transmit the force created in the muscle to the bone, making joint movement possible and allowing the maintenance of body posture. Healthy tendons possess a fibro-elastic texture that shows a great resistance to mechanical loads with minimal deformations. They appear white because they are relatively avascular and they are mainly made of extracellular matrix (ECM), based on 30% collagen, 2% elastin, and 68% water. Elastin contributes to the flexibility of the tendon, while collagen is responsible for the resistance [8]. Tendon characteristics are related to their function in the muscle–tendon complex: short and broad tendons transmit powerful and resistive forces (such as in quadriceps), long and thin tendons transmit soft and delicate movements (such as in the finger flexors). Each muscle has two tendons, proximal and distal. The muscle to tendon joint is the myotendinous junction (MTJ), while the bone to tendon joint is called the osteotendinous junction (OTJ) [8,9].

Tendons are composed of fibrous structures, responsible for their biomechanical properties, and they possess non-linear viscoelastic properties that confer rigidity combined with flexibility, allowing the force transmission from muscle to bone [10].

**Figure 1:**
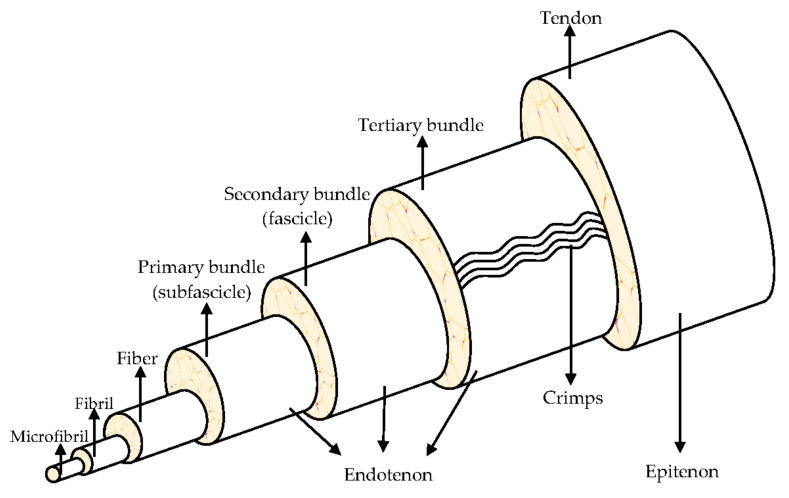
Hierarchical structure of the tendon. Modified from [9].

They are organized in a highly hierarchical structure (Figure 1) composed of collagen, which is bundled into larger progressive subunits [10,11]. The smallest tendon units are the collagen microfibril, arranged in larger fibrils from 10 nm to 500 nm in diameter. A bundle of fibrils forms a collagen fiber, which is the basic unit of a tendon, it is visible using light microscopy [8,12].

Tendons consist of closely packed collagen fibers, parallel to one another, to ensure an optimal resistance to mechanical stresses that the tendon is subjected to, during the muscular tension. The high mechanical load the tendon supports, is due to the collagen fibers alignment in the direction of load application. Tropocollagen is a triple helix type I collagen (synthetized by tendon fibroblasts and tenocytes). Five tropocollagen molecules stacked in a quarter stage array form a myofibril and neighboring myofibrils interdigitate assemble a fibril, the smallest tendon structural unit with 10–500 nm diameter. Fibers are made of collagen fibrils, having a 3–7 μm diameter. The fibers are bound together to form fascicles, that are surrounded by the endotenon, a sheath of connective tissue with nerves and blood vessels. The fascicles are distinguished in primary bundles or subfascicles, with 15–400 mm diameter, secondary bundles or fascicles, with 150–1000 mm diameter, and tertiary bundles, with 1000–3000 mm diameter. Lastly, the tendon is enclosed by the epitenon, which encircles the periphery of the tendon, and it is surrounded by the paratenon, the outermost layer. A fluid is found between the paratenon and the epitenon to prevent friction and, therefore, to both facilitate and lubricate the tendon movement [8,10,13].

The collagen metabolism in tendons is relatively slow (compared to bone, connective tissue, and articular cartilage), and a balance between its synthesis and degradation is present. This is normally increased after an injury or an exercise [12].

## 3. Tendon Cellular Component and ECM

**Figure 2:**
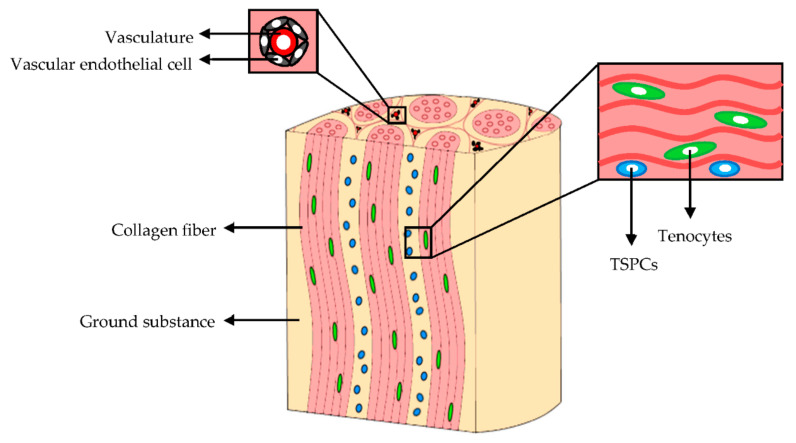
Extracellular matrix (ECM) and cellular components of tendons. Modified from [15]. CC BY 4.0.

The cellular component of mature tendons (Figure 2) is composed by several cell types although the cellular content accounts for 20% of the overall tissue volume. Tenocytes, the primary cells, represent approximately 90% of the tendon cellular compartment. They are bipolar cells with elongated nuclei and spindle-shaped morphologies; they are arranged in columns along the collagen bundles. Tenocytes have a fundamental role in tendon homeostasis since they synthesize the collagen, and contribute to the maintenance of the macromolecular components of ECM [14,15].

The remaining 10% of the cellular component are synovial cells, chondrocytes, located in proximity of the tendon-to-bone insertions, and vascular endothelial cells [10]. Moreover, tendon stem and progenitor cells (TSPCs), which show self-renewal, clonogenicity, and trilineage differentiation capacity (adipogenic, osteogenic, and chondrogenic cells), have been identified in niches within the tendon fascicles, the epitenon, and among pericytes or perivascular cells derived from the surrounding vasculature [15,16,17,18]. TSPCs exposure to mechanical stimuli of the in vivo microenvironment causes their differentiation into tenocytes or non-tenocyte cells [19,20,21,22] and regulates their proliferation [22,23]. The TSPCs gene expression of matrix proteins, integrins, and metalloproteinases during the tendon development and repair, is strongly upregulated in vitro after 3 days of mechanical stimulation [22].

Next to cell component, ECM and its hierarchical structure is of fundamental importance and it is tightly linked to tendon function. Collagen is interposed between layers of a non-collagenous matrix rich in proteoglycans, and this contributes to the tendon non-linear and viscoelastic mechanical behavior and its normal function [24]. Other than collagen, ECM is made of elastin, ground substance, and inorganic components.

Collagen is the main ECM structural protein. Collagen type I constitutes 60% of the dry mass and 95% of the total collagen, but other collagen isotypes (III, V, VI, XI, XII, XIV) are present [10]. The abundance of these is also related to the pathophysiological state of the tendon, as in the injured tendon, collagen III increases [9,25].

Elastin represents 1–2% of the total dry mass of the tendon. It is organized in fibers forming a network and provides resilience and elasticity to ECM. In fact, it contributes to the recovery of the collagen fiber wavy configuration after tendon stretch and muscle tension, and it provides flexibility and extensibility to the tissue [9,10]. In some pathological conditions (such as Ehlers–Danlos syndrome) elastin fibers increase.

Ground substance surrounds collagen and elastin, and it is mainly made of proteoglycans (PGs) and glycosaminoglycans (GAGs). Interestingly, these possess a high water-binding capacity that improves the biomechanical properties of tendons, such as elasticity against compressive forces. PGs and GAGs also stabilize the collagen hierarchical structure and maintain the ion homeostasis. In particular, PGs are composed of a core where GAGs are covalently attached, and they are entrapped within collagen fibers [24]. They confer to the tendons high capacity to withstand the forces because of their rigidity, due to the charge-to-charge repulsion, and their high charge density. In fact, PG concentration, type, and quantity depends on the tendon tensile and the compressed regions [10,26]. Other glycoproteins, such as fibronectin, tenascin C (TNC), cartilage oligomeric matrix protein (COMP), tenomodulin (Tnmd), and thrombospondin-4 (TPS4) are also important components of the tendon. They possess a low molecular weight compared to PGs. Several of these proteins are able to regulate fibrillogenesis in terms of fibril diameter, alignment, and stability. Fibronectin and TNC enhance tendon mechanical stability and allow tendons to recover the pre-stretched length after physiological loading. TPS4 is abundant in mature tenocytes, it is associated to fibrillar structure, and regulates the collagen assembly, organization, and ECM remodeling. Moreover, small leucine rich proteoglycans (SLRPs) abundant in ECM act as regulators of collagen fibrils self-assembly. Furthermore, fibromodulin, one of the most expressed in tendons, is crucial for the organization of the collagen fibrils. Besides these, tenomodulin, highly expressed in developing and mature tendon, is a marker of tenocyte differentiation, and has been recently reported as a mechanosensitive one since its expression rapidly decreases in static culture, but it is restored upon axial stretching [27,28].

Lastly, inorganic components are less than the 0.2% of the tendon dry mass. Ca^2+^ is the most present (0.001–0.01% of tendon dry weight), and in pathological conditions, its concentration may increase. Ca^2+^ has a key role in the development of the osteotendinous junction. Other ions are Mg^2+^, Co^3+^, Zn^2+^, Cu^2+^, Mn^2+^, Ni^2+^, Cd^3+^, Li^+^, F^−^, Pb^2+^, Si^2+^, and PO_4_^3−^, and they are involved in growth, development, and normal metabolism of the musculoskeletal structures [9,12,14].

## 4. Tendon-Bone Insertion (TBI) and Myotendinous Junction (MTJ)

**Figure 3:**
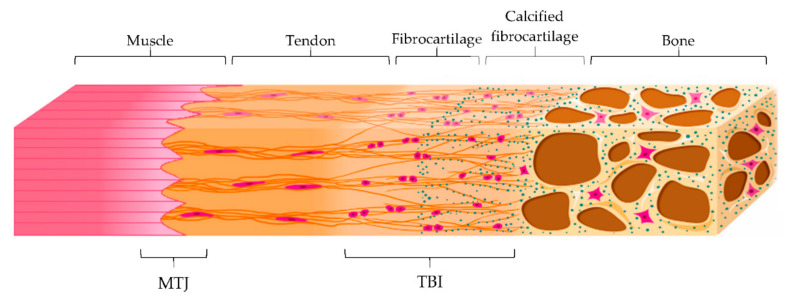
Schematic representation of the myotendinous junction (MTJ) and the tendon-to-bone interface (TBI) with its different zones: tendon, fibrocartilage, calcified fibrocartilage and bone. Modified from [30]. CC BY 4.0.

Tendon main function is the transmission of forces from muscle contraction to the bone to allow the movement, minimizing stress. Both tendon-bone insertion (TBI) and myotendinous junction (MTJ) are made of complex transitional tissue (Figure 3) that cannot be restored following injury, leading to high incidence of reoccurrence [29].

MTJ is a specialized region located at the muscle-tendon interface, representing the primary site of force transmission. Structurally, it consists of subsarcolemmal, transmembrane, and extracellular protein complexes folded into invaginations and extensions in order to increase the interface area and, therefore, the resistance of the tissue to muscle contraction forces [29].

On the other hand, TBI is the region that connects the tendon to the bone. There are two different types of TBI, depending on the type of tissue present at the attachment site: dense fibrous connective tissue or fibrocartilage one. In the first type, the insertion is characterized by a dense fibrous connective tissue, similar to the tendon midsubstance, and it attacks directly to the bone. This insertion occurs over large surface areas perforating mineralized collagen fibers and is common in tendons attached to diaphyses of large bones, such as the deltoid, which inserts into the humerus.

In the second type of TBI, a layer of fibrocartilage, as a transition from the fibrous tendon tissue to bone, is found between the tendon and the bone. In this case, the tendon bundles are directly inserted into the bone and the tendon tissue close to the bone calcifies. This type of insertion is more common and prone to overuse injuries, such as in the case of the Achilles tendon, and it is characterized by four zones that creates a continuous gradient: tendon, fibrocartilage, calcified fibrocartilage, and bone. Particularly, the fibrocartilage zone, where chondrocytes and cartilage matrix are present between collagen fibers in the tendon, acts as a shock absorber to dissipate the stress generated by bending collagen fibers in the tendon. On the other hand, the calcified fibrocartilage is an avascular and irregular zone, populated by fibrochondrocytes and consisting of type I, II and X collagen, which represents the true junction to the bone. It provides the mechanical integrity of the insertion, allowing the mechanical transition of force across the insertion [30,31].

The healing process is based on a cascade of events starting from the recruitment of monocytes and macrophages and the cytokines and growth factors release (inflammatory phase) to the angiogenesis and tenocytes proliferation and ECM synthesis (proliferative phase), to ECM maturation (remodeling and modelling phases). Tendon injuries first repair by means of the deposition of an initial matrix to form a tissue template. The cells, taking part to this early event, seem to originate from extrinsic compartment (synovium like fascias—paratenon, epitenon, and endotenon) as the resident cells possess a limited reparative capacity, being in low number and with low metabolic rate [32].

Although numerous questions remain about the formation of transitional tissues and about the enhancement of the junction healing, tissue engineering, and biological augmentation, could have a significant impact on tendon injuries outcome and clinical practice.

## 5. Tendon Mechanical Properties

The tendon mechanical properties (directly related to ECM hierarchical structure) are fundamental for their function. In the force transmission from muscles to tendons, the ratio between the strength of muscular tension and the tendon resistance to tensile force should remain constant (this should be also constant throughout life) [9] although the aging and other degenerative processes could deteriorate tendon structure [30].

**Figure 4:**
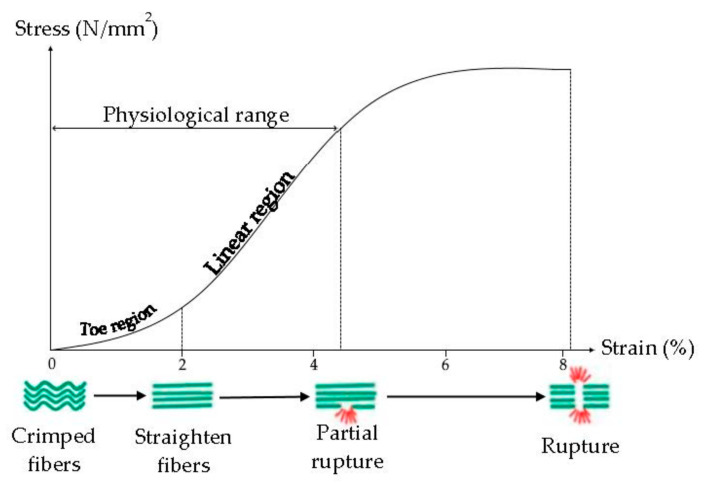
Mechanism of internal deformation of tendon. Modified from [37]. CC BY-NC-SA 3.0.

Tendon viscoelasticity, due to collagen fibers and elastin, possesses a time-dependent behavior, it is non-linear, and characterized by a Young modulus (the slope of the stress-strain curve) that increases from small values at small strains to high values at higher strains (Figure 4) [33]. Typically, the ultimate tensile strength of native tendon and ligament ranges from 5 to 100 MPa with a strain of failure 10–15% and Young’s Modulus from 20 to 1200 MPa [34].

It was demonstrated that the tendon mechanical properties are related to the collagen fibril diameter: fibrils with a small diameter are elastic and they have more resistant to the plastic flow, due to their surface area and interconnections, while fibrils with a large diameter are stronger and more resistant to tensile force, due to greater density of intramolecular cross-links. The tendon is characterized by smaller and larger fibrils, to have resistance to tensile force and resistance to the plastic flow [33,35].

Crimps are also essential for the main tendon functions, because they act as a shock absorber avoiding the tissue damage and giving the tendons the ability to absorb and transmit the tension force. The greater the stress on the tendon, the greater the crimp angle, in fact, all ruptured tendons have low crimp angle values [36].

## 6. Tendon Injuries

Tendon injuries are generally acute or chronic. Acute injuries are mainly due to traumatic damage of previously healthy tissue, caused by an external and acute trauma (bruises, stab wounds, lacerations), and they are characterized by a disarrangement of the tendon bundles and a loss of the tensile strength, which render tendons prone to rupture. On the other hand, chronic injuries are degenerative and often the result of overuse or a repetitive mechanical load. Direct trauma includes contusions and lacerations, while indirect tendon ones are usually a consequence of tensile overload [38].

Acute injuries involve a sudden external disruption of originally healthy tendon. Although such injuries often heal with acceptable recovery of function, the preinjury state is rarely fully restored after healing [5,32]. Tendon ruptures may also occur spontaneously during daily living activities, but in these cases, those are attributable to underlying accumulated tissue damage associated with degenerative tissue remodeling processes. Tendon matrix damage can stem from many sources (including acute tearing or cutting, oxidative damage, accumulation of micro-tears, or de novo generation of aberrant matrix within the tendon). Tendon injuries may ultimately result in the mechanical and biological propagation of the lesion until structural disruption and little is known regarding the accumulation of tendon damages and the coordination of the tissue remodeling up to restore [32].

Injury may appear at any point of the muscle–tendon–bone system in the weakest point of the unit. The term tendinopathy refers to several diseases that affect the TBI, the MTJ, and the tendon. These are caused by tendon overload or overuse or by intratendinous degeneration, which is commonly due to aging [39]. The tendon structural damages take place from repetitive strain and loading, particularly in activities that require power or technique.

Tendons possess a basal reparative ability, but when they are overwhelmed by repetitive and traumatic processes, damage occurs. The earliest tendon injury is tendinitis, an inflammatory event. This could be greatly debilitating, with pain, loss of muscle function, joint instability, and abnormal movements, adversely affecting the patient’s life [12,39]. Although, for decades, tendinopathy was thought as non-inflammatory in nature, modern research confirmed the presence of inflammatory cells, including macrophages and lymphocytes in chronic tendinopathy [40]. In fact, it has been hypothesized that inflammation plays a role in the early initiation of tendon pathologies.

It is conceivable that inflammation begins earlier than fibrotic and other degenerative tendon changes. Tendon injuries are accompanied by the endogenous expression of various mediators of inflammation, including proinflammatory and anti-inflammatory cytokines, and some growth factors: tumor necrosis factor (TNF)-α, interleukin (IL)-1β, IL-6, IL-10, vascular endothelial growth factor (VEGF), transforming growth factor beta (TGF-β), cyclooxygenase 2 (COX2) expression, and prostaglandin E2 (PGE_2_) [41].

Tendon inflammation is responsible of an inappropriate functionality that may increase the risk of tendon ruptures and re-ruptures [42].

Although degeneration and overuse seem to be the main causes for tendon injuries, it is unclear how different stresses induce different responses and, in many cases, the etiology remains unclear [43,44].

Tendon injuries are caused by intrinsic or extrinsic factors, also in combination, in fact, a multifactorial origin is generally found. In particular, intrinsic risk factors include demographic factors (sex, age, weight, and height), genetic polymorphisms, and local anatomical factors (leg length discrepancy, malalignment, and decreased flexibility), while extrinsic factors comprise therapeutic agents (corticosteroids, antibiotics), environmental conditions, and physical activity-related factors, including training patterns, techniques, and equipment. In acute traumas, the extrinsic factors are predominating; in chronic traumas, the combination between intrinsic and extrinsic factors is more common [12,45].

Anatomically, injured tendons show a degeneration that leads to an increase in vascularity, where blood vessels are randomly oriented and a disordered arrangement of fibers. Collagen matrix show unequal and irregular crimping, loosening, and increased waviness of collagen fibers with an increase of type III collagen (reparative collagen) [41]. Pain, edema, and inflammation are the first responses to injuries, and they are helpful in the early stages, leading to a restriction of activity and therefore of the damage.

The highest frequency of failure occurs in the over-30 age group, when tendons are more susceptible to injury due to a progressive deterioration of collagen. Moreover, elastin and proteoglycan matrix decrease, and the water content also decreases, leading to lower elasticity [45,46,47].

## 7. Traditional Approaches for the Treatment of Injuries

Tendons do not possess high regenerative potential, and they usually form scar tissue with scarce mechanical properties, increasing the risk of reoccurrence and leading to a long-term loss of fiber orientation [10]. Currently, two different approaches are used for the tendon injuries (acute or chronic): conservative, surgical, or a combination of the two. The healing time is primarily affected by the type of lesion, and the tendon involved rather than the approach considered. Whatever the method used, the treatment aims to eliminate pain, reduce inflammation, promote healing, and restore (as soon as possible) the joint function [48,49,50].

The conservative approach consists of rest, targeted exercises, cryotherapy with ice, anti-inflammatory drug therapy (nonsteroidal anti-inflammatory drugs (NSAIDs) and corticosteroids), orthopedic insoles, ultrasound, and laser therapy, to allow the tendon to heal naturally [48]. Due to the limited capacity of tendon self-healing, this approach requires long periods of treatment, a partial loss of function, and recurrent lesions. The source of the repair (intrinsic, involving matrix structure and composition, or extrinsic, involving tissue vascularity, state of inflammation, pain), is important, and a scar, mainly caused by extrinsic compartment, often results in an impaired range of joint motion. Moreover, a gap in the muscle–tendon complex could occur due to tendon retraction, and this further delays healing rate. Another key point is the extent of the wound: an extended injury (such as a total break) normally requires surgical treatment [49]. Although conservative treatment could avoid the complications related to surgery, such as infection, scar adhesion, tendon necrosis, and nerve injury, surgery has become the mainstay of therapy especially for acute injuries. The conventional surgical treatment aims to restore the tendon biomechanical properties [50,51,52,53,54,55] and involves the suturing of the wounds or the fixation of the tendon to the bone with multiple sutures, wire loops, and stainless steel anchors to the soft tissue. Both the “open air” technique and arthroscopic repair are equally considered [47]. These are often combined with the typical conservative treatments (rest, targeted exercises, cryotherapy with ice, anti-inflammatory drug therapy (NSAID and corticosteroids), ultrasound, and laser therapy) to accelerate the functional recovery (combined approach).

Tissue grafting is an alternative included in surgery approach. Autograft is the best choice to avoid tissue rejection; however, complication at the donor site could occur, and the reconstruction allows almost 50% of the ligament functions from the pre-injured state [34].

However, these approaches largely fail in presence of chronic injuries, since excessive tension could be present after the primary repair, and the failure reaches the 38%. This may occur because of tendon weakening, muscle atrophy, and contraction, decreased range of joint motion and postoperative alterations of the joint normal mechanics.

Since no consensus has yet been reached regarding the optimal treatment protocol [56], this highlights the need to find other innovative approaches to improve the repair strength [49].

## 8. New Strategies for the Treatment of Injuries: Tissue Engineering

Recently, many efforts have been attempted to find innovative approaches in tendon injuries. Tissue engineering is a multidisciplinary approach for creating a viable tissue by applying the principles and the methods of engineering and life sciences, with the ultimate aim to induce repair, and replacement or regeneration of injured tissue. Tissue engineering involves the use of cells combined to scaffolds and biologics (biological active molecules, mainly hemoderivatives and growth factors). Tissue engineered substitutes are the promising tissue replacement to overcome the limitations related to the traditional approaches although, to date, translation in clinic does not yet occur. However, in this perspective, cell-based therapy supported by scaffolds and cellular or acellular scaffolds have been proposed, both to enhance tendon regeneration and to control growth factor release to achieve tendon repair with minimal scar [48,57,58].

### 8.1. Tendon-Specific Stem and Progenitor Cells (TSPCs) Therapy

Cell-based therapy is based on direct injection of cells in the damage site: these are able to induce the in-situ production of ECM, effective in the healing process. In particular, TSPCs possess the ability to differentiate into tenocytes and into several non-tendon cell types, such as chondrocytes and osteocytes, demonstrating a tremendous potential to improve the healing of damaged tendons [57,59].

Specifically, it has been demonstrated that TSPCs are able to promote the tendon repair in a murine model of patellar tendon defect model, by increasing collagen production and by restoring collagen 3D structure, thus recovering tendon elasticity [60].

Similarly, it was stated that autologous tenocytes (a mixture of tenocytes and TSPCs) improve the histological outcomes and increase the tendon collagen content and its tensile strength, by healing chronic Achilles tendinopathy in a rabbit model [61].

Although many studies suggest that these cell therapy treatments increase the healing of the tendon injury, the translation in clinical practice needs to overcome some problems [57]. The major one is the identification of the suitable cell source and the appropriate cell number, both effective at recovering the tendon injuries. These are crucial, considering that, currently, no standard has been identified [57,62]. Along with this, the donor age seems to be another key issue [57,63].

The standardization of the therapeutic approach is a critical point, and this greatly influences the final outcome. Patient age, delivery techniques, and cell stemness, and their viability after injection or implant, are variables that should be considered. Furthermore, a carrier should be considered to prevent cell membrane breaking during the direct injection, and to facilitate cell survival and activities after implant [51]. Stem cell proliferation after implantation is an additional crucial aspect and the possible uncontrolled proliferation causes a certain degree of caution in the clinical translation. Although the use of TSPCs to improve tendon repair is an attractive option to treat diseases, such as tendinopathies, their potential clinical application is still an emerging field, and further research is required to establish a solid standard procedure to their administration.

### 8.2. 3D Scaffolds

The scaffolds, three-dimensional (3D) constructs considered the basis of tissue engineering [64,65], seem to be suitable to restore tendon injuries since they are able to recreate the spatial organization of the original tissue, to allow easier adhesion, survival, migration, proliferation, and differentiation of native cells, or seeded cells [65,66,67,68].

Resorbable scaffolds have been developed to overcome the problems arising from permanent implants and the rate of reabsorption should be planned considering the type of tissue involved. Scaffold degradation should be relatively slow to support the mechanical load until the regeneration of the new tissue. In the first phase of the repair process, the scaffold should protect the cells and the new tissue from high strains, and should allow gradual exposure to the loads in the later stages. Ideally, the system should degrade at the same speed of regeneration of the new tissue, and the degradation products should be biocompatible, without the onset of chronic inflammation or unwanted biological responses [69,70]. In particular, immune response should be negligible to prevent severe inflammatory reactions that delay the healing or cause a rejection [67,71].

Different (bio)materials have been proposed to achieve the tendon mechanical properties [48,67,71] and to resemble the tendon architecture. Porosity and pore size are key points, since cell penetration into the scaffolds and parallelly adequate nutrient diffusion should be assured. Moreover, scaffold architecture is crucial and influences the fate and the function of the implanted cells, for this reason, 3D constructs able to mimic the in vivo cell microenvironment seems critical [71].

Furthermore, during the design and the development of scaffolds, the manufacturing technology and the implant technique should be carefully evaluated to render them clinically and commercially available. Process scale-up, cost of production, and storage should be considered [67,68,69,71]. Among the various manufacturing technologies, electrospinning, soft lithography, and 3D printing have gained interest in the scientific community.

#### 8.2.1. Emerging Manufacturing Methods

##### Electrospinning

Electrospinning is a method that allows the generation of nanofibrous porous scaffolds using a high voltage electric field. It is a simple, versatile, flexible, and cost-effective method to spin polymeric materials to generate ultra-thin fibers with diameters in the nanometric or micrometric range [72,73].

**Figure 5:**
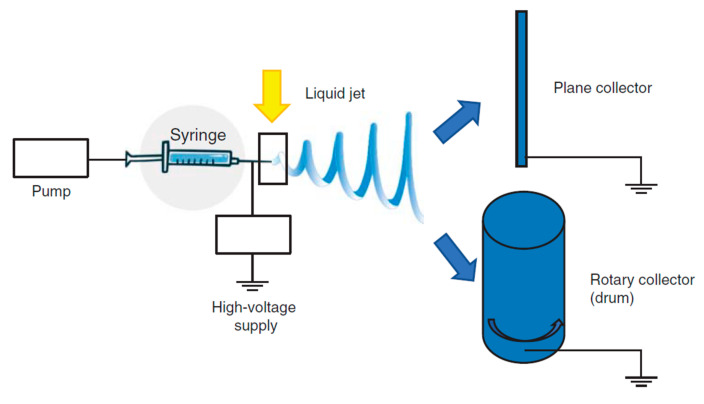
Functioning scheme of electrospinning. Modified from [72].

The basic experimental set up for electrospinning includes (Figure 5) a syringe, a spinneret (usually a needle or a capillary tube), a syringe pump, a high voltage power supply, a collector, generally planar or rotary.

The polymer solution is pumped through the syringe and it forms a droplet on the needle tip. The high voltage forces the droplet by adopting a conical shape, called “Taylor cone”, and a thin liquid jet forms as soon as the electrical field strength exceeds the solution surface tension. The liquid jet travels in spinning motions to the collector, simultaneously the solvent evaporates and the fiber is deposited onto the collector. Depending on the collector shape, such as the static or the rotating collectors, flat sheets or cylindrical structure are obtained with a great surface to volume ratio and a great porosity [72,74]. The mechanical properties are a key point and they are tuned by chemical composition and scaffold morphology (nanofiber dimension and alignment) [73,75,76,77,78] to form a 3D structure resembling tendon ECM. Nanofiber alignment obtained using a drum [71,79,80] better simulates the collagen network and this seems to favor an adequate environment for cell homing and proliferation and ECM synthesis. Morphology, combined with the high surface area to volume ratio, and mechanical properties, allow cells to maintain their phenotype and native orientation [72,81]. Moreover nanofibrous scaffolds could be easily loaded with drugs capable to enhance cell proliferation or to prevent/treat infections [82,83,84].

Aligned nanofibers based on Carbothane™ (a medical grade polyurethane) and reinforced with different percentages of multi-walled carbon nanotubes (0.06%, 0.33%, and 0.66%) demonstrated to possess better mechanical properties than random ones. Moreover, mouse embryonic fibroblasts (NIH 3T3) adhesion, and proliferation were assessed [85]. Analogously aligned fibers based on blends of poly (l-lactic acid), polyethylene oxide, and loaded with Trichostatin A, evidenced the capability to restore structural and mechanical properties of Achilles tendon in an in vivo preclinical model [86].

##### Soft Lithography

Soft lithography is an innovative strategy based on printing and molding and it represents a convenient, effective, and low-cost method for the manufacturing of micro- and nanostructures, such as scaffolds or microengineered hydrogels [87,88,89]. In soft lithography, an elastomeric stamp, with molded relief structures on its surface, ranging from 30 nm to 100 nm, is used to generate a 3D structure that reproduce the ECM [90,91,92,93].

**Figure 6:**
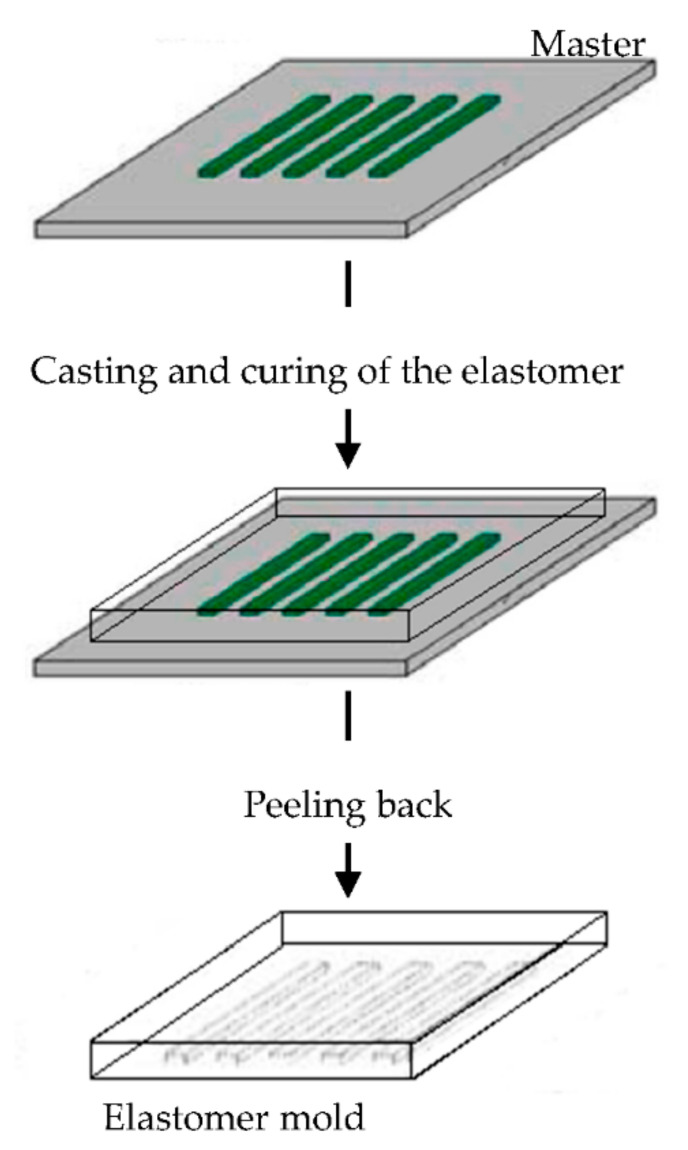
Schematic illustration of the procedure for building poly(dimethylsiloxane) (PDMS) molds for soft lithography. Modified from [94]. CC BY-NC-ND 3.0.

The elastomeric mold is fundamental in soft lithography and it is prepared by cast molding using elastomers, such as poly(dimethylsiloxane) (PDMS) or silicone rubbers, polyurethanes, and polymides. A prepolymer of the elastomer is poured over a master having relief structures on its surface, then cured and peeled off (Figure 6). The elastomers are preferentially used because they make a conformal contact with large surfaces and they are also released more easily from rigid masters or structures that are being molded [90,91,92,93,94,95,96].

Imprinted substrates with the same size range as those found in tendon has been used to investigate the relationship between the substrate topography, the tenocyte behavior, and their phenotype stability. Groove with a smaller wide than that of tenocytes promoted cell alignment, elongation, and support phenotype stability. Moreover, tenocytes with phenotype loss regained expression of tenogenic markers [97].

Different soft lithography techniques are available: microcontact printing (μCP), replica molding (REM), microtransfer molding (μTM), micromolding in capillaries (MIMIC), solvent-assisted micromolding (SAMIM) [90,91].

**Figure 7:**
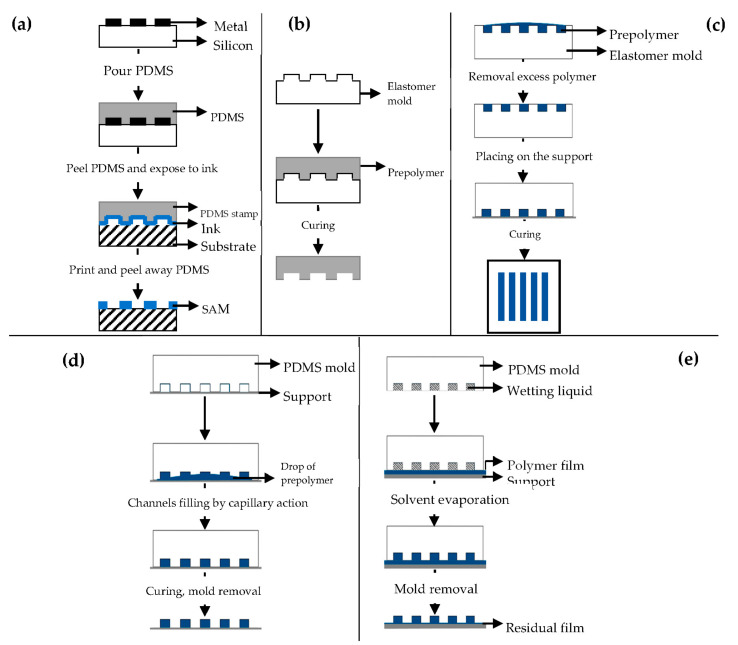
Schematic illustration of: (**a**) microcontact printing (μCP); (**b**) replica molding (REM); (**c**) microtransfer molding (μTM); (**d**) micromolding in capillaries (MIMIC); (**e**) solvent-assisted micromolding (SAMIM). Modified from [99,101]. CC BY 3.0.

μCP (Figure 7a) offers the possibility to engineer the surface properties at a molecular level [98,99]. Self-assembled monolayers (SAMs) of alkanethiols on a substrate coated with a metal, such as gold (Au), copper (Cu), silver (Ag), platinum (Pt) or palladium (Pd), form microstructures of different materials [92], and they are easily prepared by physical vapor deposition, such as thermal or electron beam evaporation [91,92].

REM (Figure 7b) is an efficient method for the duplication, in a single step, of 3D structures with shape, and morphology of the mold surface [91,98] using PDMS based molds [91,92,93,94,100]. It is based on microfluidic technique.

μTM (Figure 7c) involves a mold filled with a prepolymer and a subsequent curing process to form a solid by means of UV light or heating [91,98].

MIMIC (Figure 7d) consists of placing an elastomer mold to form a network of empty capillaries. A drop of liquid prepolymer is then placed at the open end of the capillaries spontaneously filling them by capillary action. The prepolymer is then cured into a solid and the mold is removed to leave patterned microstructures of the polymer [91,98,101,102].

SAMIM (Figure 7e) is very similar to MIMIC.

The liquid prepolymer is dissolved in a suitable solvent and not melted without the need of high temperatures [91,93,98]. When the solvent evaporates, the liquid polymer solidifies and forms a molded structure complementary to the surface of the mold.

##### 3D Printing

Three-dimensional (3D) printing is a versatile technique and scaffolds with defined shapes, controlled chemistry, and interconnected porous structures, able to mimic the ECM properties, can be manufactured [103,104,105,106,107,108].

The equipment is based on a regular inkjet print head that deposits a binder material onto a powder bed layer-by-layer [103,104]. Fused deposition modeling (FDM) coupled 3D printing has been recently developed, and in this, a fused filament is used as ink. Almost all types of materials, such as polymeric and composite materials (ceramic and metallic based), have been used [109,110].

The 3D printable models are generally created with a computer-aided design (CAD) package, via a 3D scanner, or by a plain digital camera and photogrammetry software, and these methods allow to realize complex 3D structure with highly organized hierarchical organization. The 3D scaffolds based on polycaprolactone (PCL) have been built to mimic tendon hierarchical structure in fiber to fascicle, organization, allowing the attachment and alignment of the human tenocytes in vitro [111]. Moreover reinforced geometry have been obtained, as in the case of acrylonitrile butadiene styrene matrices (ABS) in collagen scaffold, without changing scaffold bioactivity [112].

#### 8.2.2. Materials

Numerous synthetic and natural materials have been considered for tendon tissue engineering.

Synthetic polymers are very attractive candidates as they are characterized by remarkable mechanical properties, and thanks to their chemico-physical properties, they are easily worked using emerging manufacturing technology as electrospinning and 3D printing. Moreover PCL (poly (-caprolactone), PLLA (poly-l-lactic acid), PLGA (poly (lactic-co-glycolic) acid), or poly urethanes (PUs) present tunable and reproducible mechanical and chemical properties (such as degradation) in spite of a marked hydrophobicity, and are biocompatible and non-toxic. Despite this, they possess limited capability to enhance cell adhesion and homing. On the contrary, natural polymers, such as collagen, gelatin, silk proteins (fibroin and sericin) alginate, chitosan, hyaluronan, are characterized by high hydrophilicity and superior bioactive properties, although it is difficult to obtain nanotopographic and reproducible structures and suitable mechanical properties. Considering this, natural polymers are often blended to synthetic polymers to obtain highly resistant systems able to interact with cells. This is the fundamental step toward intracellular signal stimulation to regulate cell migration, cell proliferation, and cell differentiation. Moreover, it has been proved that surface hydrophilicity or hydrophobicity could induce conformational changes of integrin-binding proteins, resulting in different adhesion strength [113].

## 9. Biological Augmentation for Tendon Healing

Besides the development of 3D hierarchical scaffolds, the use of biologicals is currently under investigation to enhance the scaffold performance without scar formation. Recently, various biologics combined with scaffolds have been considered and evaluated in preclinical animal studies to augment tendon repair [114,115,116]. The main goal is to achieve a complete functional recovery of the tendon injuries [10,117].

Recombinant growth factors (GFs) have been considered and nanoencapsulation was used as strategy to increase their stability and control their release. GFs are normally involved in the healing process and play an important role in the regulation of the tendon healing phases, since they tune cell proliferation, differentiation, chemotaxis, and matrix synthesis [109,118,119,120,121,122,123]. Nowadays, the use of hemoderivatives have been explored for tissue engineering applications as a simple and cost-effective source of GFs, cytokines, and structural proteins, which possess a key role in the regeneration of bone and soft tissues. In fact, the combination of different GFs is expected to have a synergistic effect on the regulation of tissue regeneration [124,125].

Despite the over 1600 clinical trials registered con ClinicalTrials.gov for the use of GFs, widespread regulatory approval and marketing are far from coming. Only a few, single GF-based formulations have been approved, by US-FDA (Food and Drug Administration) bone morphogenic protein (BMP)-2 and BMP-7 for lumbar spine fusion and bone fracture, platelet derived growth factor in BB isoform (PDGF)-BB for enhancement of granulation tissue formation and keratinocyte growth factor (KGF) for the prevention and treatment of mucositis in cancer patients; and by Japan and China, fibroblast growth factor (FGF)-2; and by Latin America and part of Asia, epidermal growth factor (EGF), all for wound healing [126].

In fact, hemoderivatives are rich in both GFs and cytokines: they provide multiple signals required to complete the regeneration process, all fundamental to modulate and eventually accelerate the healing process. Moreover, hemoderivatives may contain β-lysin with antimicrobial properties [127,128,129] and structural proteins, such as fibrinogen and fibronectin which act as matrix for cell adhesion and migration [130].

**Figure 8:**
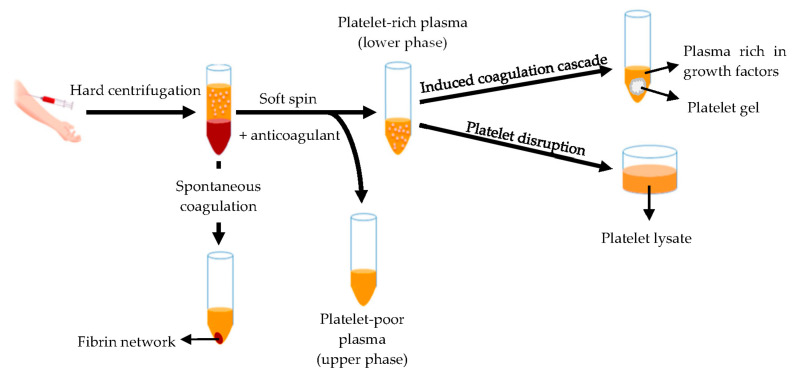
Schematic representation of the blood derivatives production by means of double centrifugation technique. Modified from [141]. CC BY 4.0.

Hemoderivatives are prepared starting from whole blood or apheresis. The fastest production of blood derivatives involves the separation of blood components by means of the double centrifugation technique (Figure 8) to produce two different fractions: the platelet-poor plasma and the platelet-rich plasma (PRP) [131,132].

The platelet-rich plasma is defined as a volume of plasma that contains a platelet concentration above blood normal baseline (15–35 × 10^4^/μL), with an average of 20 × 10^4^/µL [132,133]. Autologous origin renders it inherently safe and free from transmissible diseases, such as HIV and hepatitis. Moreover, it can be used as liquid formulation or as platelet gel [132,134,135,136,137,138,139,140]. Otherwise platelets are disrupted originating the platelet lysate [141,142,143] rich of bioactive components involved in tissue regeneration, such as platelet derived growth factor (PDGF), epidermal growth factor (EGF), platelet derived epidermal growth factor (PDEGF), vascular endothelial growth factor (VEGF), transforming growth factor beta (TGF-β), fibroblast growth factor (FGF), insulin-like growth factor (IGF), interleukin 8 (IL-8) and tumor necrosis factor (TNF)-α [58,59,60,61,62,63,64,65,66,67,68,69,70,71,72,73,74,75,76,77,78,79,80,81,82,83,84,85,86,87,88,89,90,91,92,93,94,95,96,97,98,99,100,101,102,103,104,105,106,107,108,109,110,111,112,113,114,115,116,117,118,119,120,121,122,123,124,125,126,127,128,129,130,131,132,133,134,135,136,137,138,139,140,141,142,143,144,145,146]. Platelet lysate provides various advantages for therapeutic applications: it is liquid since the clot and platelet are removed, its production is easy to standardize, and it can be frozen and stored to be quickly available for use [142].

In recent years, hemoderivatives for tendon healing have been administered via direct injection: in situ gelling is led to tissue collagen and thromboplastin, leading to gel formation, or coagulation cascade induction, thus forming in vivo a fibrin matrix-based scaffold that mimics the natural healing process [147]. However, despite the numerous positive reports on the use of blood derivatives in the tendon regeneration field, the literature frequently shows contradictory results, and it is complicated to establish a clear cause-effect relationship due to the complexity of the formulations, showing that the development and the standardization of new strategies is of paramount importance to overcome these limitations.

Despite this, clinical evidence suggests the effectiveness of hemoderivatives in improving tendon activity, reducing pain and, lastly, tissue reparation [131].

Recent clinical trials have shown midterm positive outcomes (improved activity level and reduced pain) on multiple platelet rich plasma injections for the treatment of various tendinopathies as Achilles and rotator cuff. In the first one [148], eight studies were considered and the clinical outcomes of rotator cuff repair with and without platelet rich plasma were analyzed, while in the second one (eight studies) [149], PRP was compared to steroid treatment on lateral epicondylitis. Both the meta-analyses suggest that platelet rich plasma was effective in reducing pain and improving tendon function in the intermediate term (12 weeks), and long-term (1 year), although there were no particular differences in tissue integrity [150].

However, newer meta-analyses aimed to evidence the effectiveness of platelet-rich plasma treatments in tissue healing. In the first one [151], 11 studies (randomized controlled trials) compared 355 patients treated with PRP at the tendon-bone interface to 351 patients treated with a control in rotator cuff repair, as they reported the tendon healing rate. A statistical difference in favor of PRP was reported, in fact, with PRP, 17.2% of patients showed incomplete tendon healing, while in the control, 30.5% of patients showed incomplete tendon healing. The difference was statistically in favor of PRP, both in small-medium and medium-large tears, demonstrating that PRP possesses clinical benefits in improving the tendon healing rates of all tears, independently of their dimensions. It also significantly reduces pain levels in the immediate postoperative period and increases the functional outcomes when compared with a control. In the second meta-analysis of randomized controlled trials [152], the efficacy of PRP applied to the tendon-bone interface in the arthroscopic rotator cuff repair was also demonstrated. It considered seven studies, which included 233 patients treated with PRP and 231 patients treated with a control, which reported the retear rate, which was significantly lower in the PRP group compared to the control. Moreover, these studies reported the efficacy of PRP in improving the functional outcomes, especially in the short-term.

However, the absence of standardization of the preparation methods, such as the donor number and the anticoagulant agents involved, generally leads to considerable differences in the composition and concentration of the blood derivatives. For this reason, the preparation is a key point to control and accomplish the desired therapeutic effect [135]. Another key point is the GF concentration (effective and safe concentration and ratio between different factors) and delivery rate at the target, since in vivo ECM regulates GF availability and signaling [153,154]. This highlights the need of a standard method for hemoderivative administration through the conjugation of scaffolds able to mimic the ECM matrix.

Furthermore, scaffolds should stabilize GFs from external stress, light, temperature, pH and ionic strength that may induce protein conformational change and, consequently, reduce their biological activity [155,156].

Table 1 reports examples of the in vivo studies carried out so far on scaffolds combined with biologics for tendon repair, underlying the possible advantages in the treatment of tendon pathologies.

## 10. Conclusions and Future Perspectives

Chronic, non-healing tendon injuries frequently require surgical treatment, and despite recent advancements in orthopedic surgery, they present several limitations with a high (38%) percentage of failure, thus leading to the loss of the tendon and joint correct function.

Nowadays, in a biomimetic regenerative medicine approaches, an important challenge is the development of 3D scaffolds with hierarchical structure for the control of the spatiotemporal and selective delivery of blood derivatives, and many attempts have been made to identify suitable carriers to deliver GFs in the injury site. This strategy should increase their therapeutic effects, enhancing healing and leading to the formation of a functional tissue without scar formation [109]. Biomaterials have a crucial role: their intrinsic properties may increase the controlled delivery of hemoderivatives and may synergistically potentiate the healing process [166,167,168]. The control of the spatial-temporal release profiles should potentiate the effectiveness of future treatments [117], avoiding GF degradation and uncontrolled cell proliferation growth, and prolonging GFs short half-life in vivo [156,169,170,171,172].

Innovative strategies focus on the development of scaffolds to enhance tendon restore. Electrospinning, soft lithography, and 3D printing are emerging techniques used to manufacture 3D scaffolds with suitable geometry and structure to replace damaged tissue and support tendon reparation. Although great advancements have been achieved in manufacture techniques, biomaterials or biomimetic structures are not enough performing to efficiently restore tendon and joint functions. Many attempts have been made to enhance scaffold effectiveness and the biological augmentation seems the more convenient option to obtain both tissue healing and fast translation towards clinic.

Healing of the tendon-to-bone interface remains a challenge due to the peculiar anatomy and structure, and the association of inorganic materials with biologics in 3D scaffolds should be a strategy to both enhance hard/soft tissue interface.

Current research activities point toward finding an optimal 3D structure with suitable mechanical properties, capable of delivering biologics, having a synergic response on tendon recovery.

## Figures and Tables

**Table 1:** In vivo studies using growth factors (GFs) and blood derivatives for tendon repair.

Hemoderivatives	Carrier System	Anatomical Site	Model	Main Outcomes
TGF-β1	Alginate scaffold	Bilateral supraspinatus tendon	Rabbit [157]	More evident formation of fibrocartilage; Better collagen orientation, organization, and continuity; Increase of the ultimate failure load compared to the non-treated groups.
TGF-β3	Heparin/fibrin-based system	Supraspinatus tendon	Rat [158]	Initial increase of proliferation, cellularity, inflammation and vascularity; Subsequent improvement of ultimate force, modulus, failure to stress and toughness.
bFGF	PLGA fibrous membranes	Rotator cuff	Rat [159]	Membrane absorption in 2 weeks; Increase of collagen organization and formation of a more mature tissue in compare to groups treated with PLGA membrane alone; Increase in cross-sectional area and consequent reduction of the ultimate stress; Increase of the ultimate load to failure.
FGF-2	Fibrin sealant	Supraspinatus tendon	Rat [160]	Increase in cellularity and vascularity at the tendon-to-bone interface; Increase of the parallel-oriented fibers, bone ingrowth and strength compared to the non-treated group in 2 weeks; Treated and non-treated groups manifested similar strength related to the tendon-to-bone interface maturity in 4 weeks.
GDF-5	Suture	Achilles tendon	Rat [161]	Increase of the tensile strength and maximum failure load in 2 weeks; Increase of the thickness and cell density; Appearance of cartilage-like cells in 4 weeks.
BMP-7	Gelatin hydrogel sheet	Rotator cuff	Rat [162]	At the tendon-to-bone interface, increase of the number of chondrocytes, higher maturity and ultimate load to failure; Higher orientation of the collagen fibers; Higher deposition of collagen matrix compared to the groups treated with BMP-7 alone.
rh-BMP-2	Dermal patch	Rotator cuff	Rabbit [163]	Increase of the ultimate tensile strength and of new bone formation compared to the groups treated with suture and dermal patch alone; Higher cellularity at the tendon-to-bone interface and presence of new fibrochondrocytes.
PRP	Local injection	Achilles tendon	Rat [164]	Formation of a collagen fibers transition zone and increase of the mechanical strength; Proteoglycan expression; Complete healing compared to the non-treated groups.
PRP	Hamstring tendon grafts	Anterior cruciate ligament	Rabbit [165]	Formation of mineralized tissue, new bone and cartilage at the tendon-to-bone interface; Formation of aligned connective tissue.

Abbreviations: TGF-β1/3, transforming growth factor-β1/3; bFGF, basic fibroblast growth factor; PLGA, poly(lactic-co-glycolic acid); FGF-2, fibroblast growth factor 2; GDF-5, growth differentiation factor 5; BMP-7, bone morphogenetic protein 7; rh-BMP-2, recombinant human bone morphogenetic protein 2; PRP, platelet-rich plasma.

## Data Availability

Data sharing not applicable.
